# Memantine combined with environmental enrichment improves spatial memory and alleviates Alzheimer's disease-like pathology in senescence-accelerated prone-8 (SAMP8) mice

**DOI:** 10.7555/JBR.26.20120053

**Published:** 2012-10-15

**Authors:** Jingde Dong, Mi Zhou, Xiaoqiang Wu, Mingyang Du, Xiaoshan Wang

**Affiliations:** a Department of Geriatric Neurology;; b Department of Neurology, Nanjing Brain Hospital Affiliated to Nanjing Medical University, Nanjing, Jiangsu 210029, China.

**Keywords:** memantine, environmental enrichment, SAMP8 mouse, spatial memory, Alzheimer's disease (AD)

## Abstract

Memantine is a *N*-methyl-*D*-aspartate (NMDA) receptor antagonist approved for the treatment of moderate to severe Alzheimer's disease (AD). Environmental enrichment (EE) has shown significant beneficial effects on functional improvement in AD. In this study, we sought to determine whether combining these two distinct therapies would yield greater benefit than either drug used alone. We investigated the effect of memantine combined with EE on spatial learning and memory and AD-like pathology in a widely used AD model, the senescence-accelerated prone mice (SAMP8). The SAMP8 mice were randomly assigned to enriched housing (EH) or standard housing (SH), where either memantine (20 mg/kg) or saline was given by gastric lavage once daily continuously for eight weeks. Our results showed that, when provided separately, memantine and EE significantly improved spatial learning and memory by shortening escape latencies and increasing the frequency of entrance into the target quadrant. When combined, memantine and EE showed additive effect on learning and memory as evidenced by significant shorter escape latencies and higher frequency of target entrance than either drug alone. Consistent with the behavior results, pathological studies showed that both memantine and EE significantly reduced hippocampal CA1 neurofibrilliary tangles (NFTs) as well as amyloid beta precursor protein (APP) levels. Combining both therapies synergistically lessened NFTs and APP expression compared to either drug alone in SAMP8 mice, indicating that the combination of memantine with EE could offer a novel and efficient therapeutic strategy for the treatment of AD.

## INTRODUCTION

Alzheimer's disease (AD) is a common neurodegenerative disorder characterized by progressive impairments in memory, language, visuospatial cognition and behavior[1]. These clinical manifestations are caused by neuron degeneration with deposition of amyloid plaques and formation of neurofibrillary tangles (NFTs), which lead to progressive and irreversible loss of neurons and cognitive decline[2]. Amyloid plaques consist of fibrils that are formed from the amyloid-β (Aβ) peptide. Aβ peptides, which primarily consist of a short peptide of 40 amino acids in length Aβ (1-40) and a longer peptide of 42 residues Aβ (1-42). are the proteolytic cleavage products of a transmembrane protein, amyloid β precursor protein (APP)[3],[4]. NFTs are intraneuronal protein aggregates arranged in paired helical filaments, formed by hyperphosphorylation of the microtube-associated protein tau.

Memantine is a uncompetitive *N*-methyl-*D*-aspartate (NMDA) receptor antagonist which is thought to selectively block the excitotoxic effects associated with abnormal glutamatergic transmission, while allowing physiological transmission associated with normal cell functioning[5]. Clinical trials have shown that memantine is an effective, safe and well-tolerated treatment option for moderate to severe AD[6],[7]. It has been shown to delay cognitive decline in patients with dementia[7]–[9], possibly by blocking the excessive influx of calcium ions through the channel of activated NMDA receptors[5]. Cellular studies indicated that memantine is a moderate affinity, uncompetitive and reversible NMDA receptor antagonist[10], and is assumed to leave physiological NMDA activation intact. One very recent study reported actions of memantine beyond NMDA receptor antagonism, including stimulatory effects on cholinergic signaling via muscarinic receptors[11]. These interactions with the cholinergic system are likely to contribute to the therapeutic potential of memantine in AD. However, a very recent clinical study reported that, despite its frequent off-label use, evidence is lacking for a benefit of memantine in mild AD. There is meager evidence for its efficacy in moderate AD[12], indicating that it is necessary to use combined or adjunctive therapies to additively or synergistically attenuate the deleterious cognitive decline in mild to moderate AD.

It is evident that rodents housed in environmental enrichment (EE) significantly alter brain biochemistry, synaptic morphology and neuronal function compared with standard-housed animals[13]. These alterations likely provide cognitive improvements after EE. Recent studies have shown that EE mitigates cognitive deficits in animal model of AD with the mechanism of enhancing cellular plasticity, reducing deposit of Aβ peptide and preventing the formation of NFTs[14]–[16].

In this study, for the first time, we proposed to evaluate the potential additive benefit of memantine and EE, both of which have shown significant therapeutic effect on AD, on spatial learning and memory as well as on AD-like pathology including NFTs and APP expression in the hippocampus in senescence-accelerated prone (SAMP8) mice.

## MATERIALS AND METHODS

### Animals

SAMP8 mouse strains were originally developed from the AKR/J strain of mice in the laboratory of Professor Toshio Takeda in Kyoto, Japan[17]. All SAMP8 mice used in this study were provided by the Experimental Animal Center of Hebei Province (Shijiazhuang, Hebei, China). All experimental procedures performed in accordance with the protocols were approved by the local Institutional Animal Care and Use Committee and were conducted at an Association for the Assessment and Accreditation of Laboratory Animal Care-approved facility.

### Experimental design

At 6 months of age, SAMP8 mice were conveyed to the experimental housing conditions. Forty male SAMP8 mice were divided in four groups and held in standard housing (SH), while another 40 male SAMP8 mice were housed in equally composed groups in enriched housing (EH). SH mice were housed in standard laboratory polycarbonate cages (30 cm×20 cm×15 cm) including sawdust as bedding material. EH mice, however, were housed in larger cages (50 cm×37 cm×40 cm) within which the animals gained access to different cognitive and physical stimulation objects, including two running wheels, a long tunnel, a few toys with different shapes and colors which were rearranged and exchanged every two days. To determine the effect of memantine combined with enriched environment on SAMP8 mice, we treated 20 of the mice within either the SH or EH group with memantine [20 mg/(kg·d)] through gastric lavage for continuous 8 weeks while the remaining 20 mice within either the SH or EH group were treated with the same doses of saline in the same way. The dose of memantine used in this study was decided according to Dong et al.[18]. Both SH and EH mice were exposed to 12 h light/12 h dark cycle (lights on at 7 a.m., off at 7 p.m.). All the behavioral experiments in this study were conducted between 9 a.m. and 3 p.m.. Food and water were provided ad libitum.

### Spatial learning and memory test

The standard Morris water maze task measures spatial learning and memory[19]. For this task, a circular stainless steel pool of 120 cm in diameter and 50 cm in height was divided into four quadrants which were marked with a triangle, square, diamond and circle, respectively. Ink staining water was filled into pool and the temperature was set at 26±1°C. Transparent escape platform (14 cm in diameter and 20 cm in height) was placed 1.5 cm below the surface of water in the middle of one of the quadrants (goal quadrant). The mice performed four consecutive trials each day over a 4-d training period. Each mouse was released and faced the wall of the maze. From the four different quadrants, we respectively recorded the escape latency of the mouse (in s), which was the time that mouse spent on finding out and climbing up the platform. If the mouse could not find out the platform in standard time (90 s), a laboratory technician would help it to climb up the platform and the mouse may go on to the next experiment after 30 s rest in the platform. In this case, the latency was recorded as 90 s. On d 5 of testing, a 90 s probe test was performed to evaluate spatial memory retention. The platform was removed from the pool and all the mice were then placed in the quadrant opposite that from which the platform had been removed and allowed to swim freely. For probe test, a circular area (three times the platform diameter) surrounding the former location of platform was delimited and used as the counting zone for establishing successful memory retention. The number of times that an animal swam into the counting zone (frequency of entrance) was recorded during the test. Moreover, the swim speed and trail was recorded at the same time.

### Immunohistochemistry

Five mice in each group (the SH-memantine treated group, the SH-saline treated group, the EH-memantine treated group and the EH-saline treated group) were anesthetized with chloral hydrate and perfused with 0.9% NaCl solution and 4% paraformaldehyde in 0.1 mol/L phosphate buffer. The brains were dissected out and immersed in 4% paraformaldehyde in 0.1 mol/L PBS for 4 h before they were transferred to 25% sucrose in PBS. After the brains sank to the bottom, coronal sections were cut (10 µm) and mounted in the slide. Bielschowsky's silver staining was performed according to a modification by Tago[20]–[22]. Slide-mounted tissue sections were placed in 20% silver nitrate for 15 min in the dark. After addition of ammonium hydroxide to the silver nitrate solution until the developing precipitate disappeared again, slides were placed in the ammoniacal silver nitrate for another 15 min in the dark. The slides were then transferred into ammoniacal distilled water. After 0.1-0.2 mL developer (containing 20 mL formalin, 0.5 g citric acid and 0.5 mL of nitric acid in 100 mL distilled water) was added to the solution of ammoniacal silver nitrate, slides were placed into this solution and developed until the required intensity of staining appeared. Slides were rinsed in tap water for 10 min, dehydrated and put on with coverslips. In Bielschowsky-stained sections, images from eight 10X-objective fields from hippocampal CA1 areas of high NFT densities were captured. The number of NFTs in each image was subsequently counted by one investigator (JD) in a blinded fashion.

### Western blotting assays

The remaining mice in each group were also anesthetized and the hippocampuses were dissected immediately and tissues were then homogenized for Western blotting analysis. The protocol was desribed previously[23]. Briefly, protein (25 µg) extracted from the cortex and hippocampus was loaded into 10% SDS-polyacrylamide gels and run for 1.5 h at 110V. Blots were then transferred to nitrocellulose membranes and blocked in 2% milk/tris-buffered saline with Tween-20 (TBS-T). Membranes were then incubated with mouse anti-APP (N-terminus) antibody (clone 22C11) with a dilution of 1:2,000 (Millipore, Billerica, MA, USA) overnight at 4°C in 1.5% milk/TBS-T followed by horseradish peroxidase-conjugated goat anti-mouse secondary antibodies (1:5,000; Jackson Immuno Research, West Grove, PA, USA) for 2 h. The immunoblots were developed with enhanced chemiluminescence (ECL; Pierce, Rockford, IL, USA). After developing, membranes were stripped and re-probed with polyclonal antibody against β-actin 1:5,000, Sigma (β-actin; Sigma, St. Louis, MO, USA) in 5% milk/TBS-T. All values were normalized to β-actin. Densitometry was performed with NIH Image J Version 1.63 (Immage J; NIH, Bethesda, Maryland, USA).

### Statistical analysis

Statistical analyses were performed using SPSS version 16.0 (SPSS Inc., Chicago, IL, USA). Data were expressed as mean±SEM. The swim speed in the spatial learning task, the differences of latencies to the target platform, the number of entries into the target quadrant during the probe test in the water maze and the NFT counts in the hippocampal CA1 region and hippocampal APP expression level were all analyzed by a two-way ANOVA with repeated measures followed by Bonferroni post-test.

**Fig. 1:**
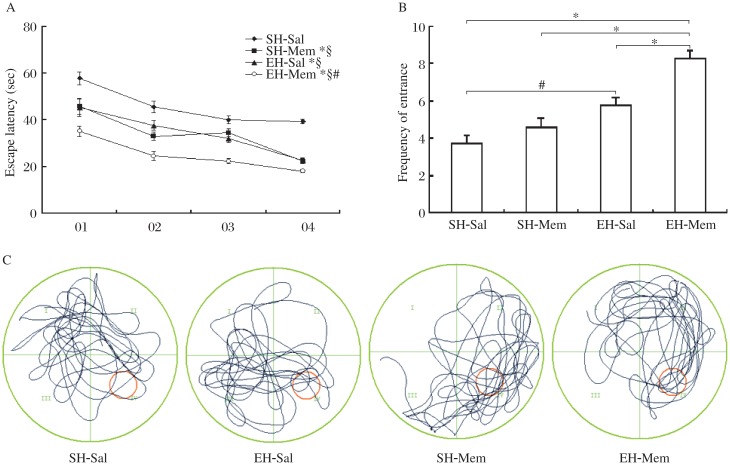
Memantine (Mem) combined with environment enrichment (EE) significantly improved spatial learning and memory in SAMP8 mice. A: Compared to standard housing (SH)-saline (sal) mice, the enriched housing (EH)-sal, SH-mem and EH-mem mice all showed significant lower latencies and a more pronounced decrease in latency during the fourth session (*P < 0.05). EH-sal, SH-mem, and EH-mem mice showed a progressive decrease in latency for finding the platform in the water maze test from the first to the fourth session (^§^*P* < 0.05). EH-mem mice had a significantly lower latency than that of SH-mem mice on the first session and EH-sal mice on the second session (^#^*P* < 0.05). B: EH-mem mice had a significantly higher frequency entering the counting region than the SH-sal, SH-mem and EH-sal mice (*P < 0.05). EH-sal mice had a significantly higher frequency of entering the counting region than SH-sal mice (*P < 0.05). However, no significant difference was observed between SH-mem and SH-sal mice and between SH-mem and EH-sal mice. C: The swim trajectories of mice in the four groups were recorded during the probe test. EH-mem mice swam mainly in the target quadrant and EH-sal and SH-mem mice showed tendencies to approach the target quadrant. However, SH-sal mice swam equally in the four quadrants.

## RESULTS

### Spatial learning and memory test

The mice within the four groups (SH-saline, EH-sal, SH-mem, and EH-mem) showed similar swimming speed (SH-sal: 17.88±2.05 cm/s; EH-sal: 18.75±1.85 cm/s; SH-mem: 18.62±1.90 cm/s; EH-mem: 18.24±2.02 cm/s), and no statistically significant differences were observed among the groups (F_1,76_ = 1.78; *P* > 0.05) and sessions (F_1,76_ = 2.15; *P* > 0.05). EH-mem mice, however, performed better than SH-mem and EH-sal mice in spatial learning task, as shown by their lower latency in finding the platform during the training sessions and the decrease in the latency in finding the platform across the four successive sessions. When compared to SH-sal mice, EH-sal mice, SH-mem mice and EH-mem mice all showed significant shorter latencies and more pronounced decrease in latency during the fourth session in comparison with that of the first session. Statistical analysis with two-way ANOVA confirmed that the latency in finding the platform had a significant effect of group (F_3,12_ = 6.27, *P* < 0.001) and session (F_3,12_ = 8.46, *P* < 0.001) and a significant interaction between group and session (F_3,9_ = 2.75, *P* < 0.001). Multiple Bonferroni test showed that the latency of EH-mem mice was significantly lower than that of SH-sal mice (through all the four sessions, *P* < 0.01), SH-mem mice (on the first session, *P* < 0.01) and EH-sal mice (on the first session, *P* < 0.05). However, the latency between SH-mem and EH-sal mice showed no significant difference during all the four sessions (*P* > 0.05) (***Fig. 1A***). The mice in the EH-mem group with best performance in learning test, which was proved by the lowest latency to find the platform, also had a greater frequency of entrance into the counting zone during the probe test. Statistical analysis indicated that the frequency of entrance into the counting zone had a significant effect of memantine treatment (F_3,28_ = 23.15, *P* < 0.001) as well as EE (F_3,28_ = 21.68, *P* < 0.001) and a significant interaction between memantine treatment and EE (F_3,9_ = 3.26, *P* < 0.001). Multiple Bonferroni test showed that EH-mem mice entered this zone more frequently (8.25±0.44) than the other three groups (EH-sal mice: 5.8±0.41; SH-mem mice: 4.6±0.50; SH-sal mice: 3.75±0.44). EH-sal mice had a significant higher frequency entering the counting region than the SH-sal mice (*P* < 0.05). However, no significant difference of frequency was observed between mice in SH-mem and SH-sal (*P* > 0.05) as well as SH-mem and EH-sal groups (*P* > 0.05) (***Fig. 1B***). Accordingly, swim trajectories of the mice in the four different groups, which are shown in ***Fig. 1C***, were recorded during the probe test. Mice in the EH-mem group swam mainly in the target quadrant and mice in EH-sal and SH-mem showed tendencies to approach the target quadrant. However, the SH-sal group swam rather equally in the four quadrants (***Fig. 1C***). Altogether, these data suggested that both memantine and EE showed significant improvement of spatial learning and memory in the senescence-accelerated prone mice. Furthermore, memantine in combination with EE yielded more benefit on learning and memory than either alone.

**Fig. 2:**
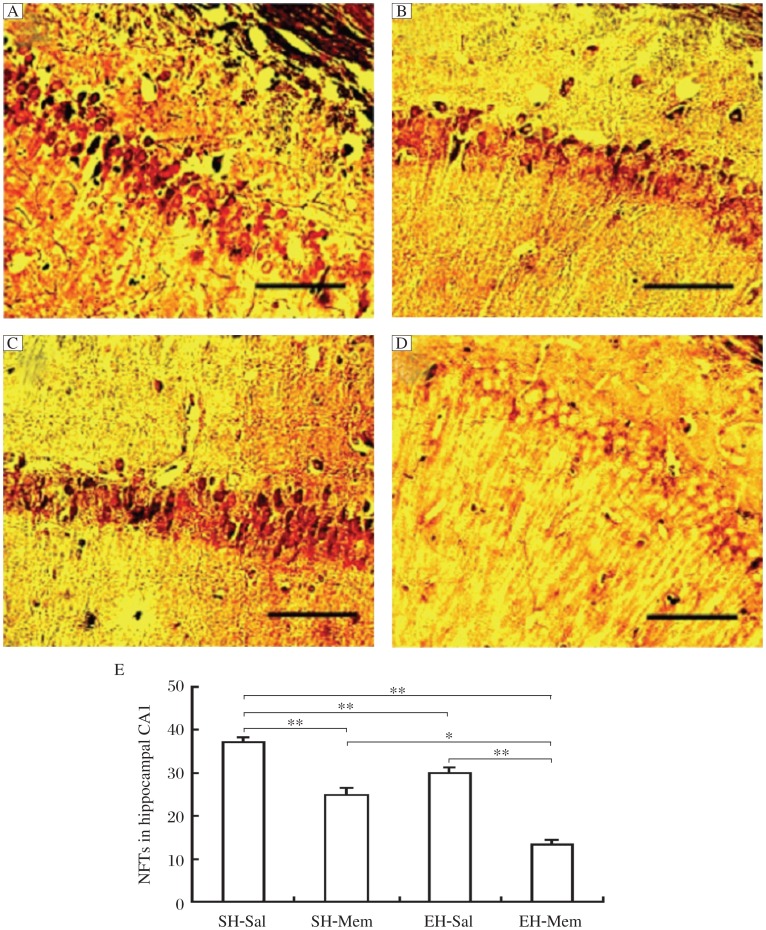
Memantine combined with environment enrichement (EE) significantly decreased neurofibrillary tangles (NFTs) in the hippocampal CA1 region in SAMP8 mice. A: Representative images of NFTs staining in Bielschowsky silver impregnated sections in SH-sal, SH-mem, EH-sal, EH-mem treated mice (scale bar=100 µm). B: Quantification of NFT aggregation in the CA1 region in the four groups of mice. Mice in all the SH-mem, EH-sal and EH-mem had significantly fewer number of NFTs in hippocampal CA1 compared to the mice in the SH-sal control group (**P < 0.01). The number of NFTs in EH-mem mice was significantly lower than that of SH-mem (*P < 0.05) and EH-sal mice (**P < 0.01); however, no significant difference was observed between the SH-mem and EH-sal mice.

**Fig. 3:**
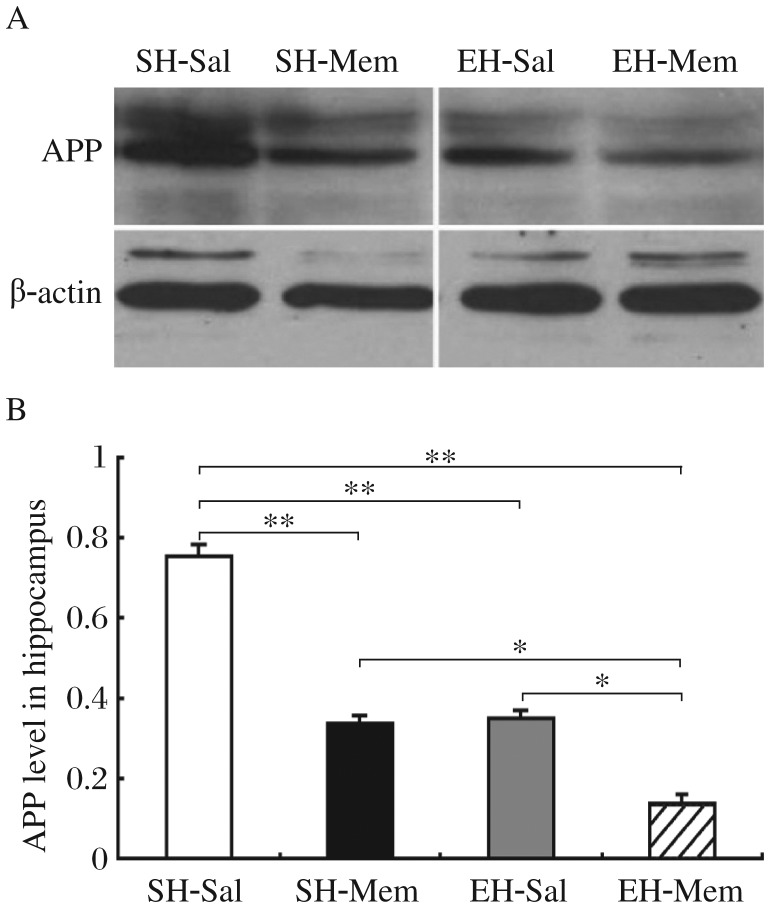
Memantine combined with environment enrichment (EE) significantly decreased APP expression in the hippocampus of SAMP8 mice. A: Western blots were probed with anti-APP antibody using protein samples from the hippocampus of SH-sal, SH-mem, EH-sal, EH-mem treated mice, respectively. B: Bar graph shows APP protein quantification in the hippocampus of different groups of mice. Mice in SH-mem, EH-sal and EH-mem had significantly lower levels of APP in the hippocampus as compared to mice in the SH-sal control group (**P < 0.01). The APP level in EH-mem mice was significantly lower than that of SH-mem (*P < 0.05) and EH-sal mice (*P < 0.05); however, no significant difference was observed between SH-mem and EH-sal mice.

### Immuohistochemistry

The staining of NFTs in Bielschowsky silver impregnated sections was performed to investigate the effect of memantine combined with EE on AD-like neuropathology in SAMP8 mice. In AD, NFTs are initially aggregated within vulnerable neurons, and eventually kill these neurons. Neurons in hippocampal CA1 are reported to be particularly vulnerable to NFT aggregation[24]. Therefore, in this study, we focused on examining NFTs in the CA1 region. Our results showed that there were many NFT aggregations characterized by dark staining in the CA1 region in SH-sal mice; however, fewer aggregations were observed in SH-mem and EH-sal mice. In EH-mem mice, very few NFTs were observed (***Fig. 2A***). Two-way ANOVA analysis indicated that NFT aggregation in the CA1 region had a significant effect of memantine treatment (F_1, 36_ = 18.27, *P* < 0.001) as well as EE (F_1,36_ = 20.34, *P* < 0.001) and a significant interaction between memantine treatment and EE (F_2,8_ = 6.52, *P* < 0.001). Multiple Bonferroni test showed that mice in SH-mem, EH-sal and EH-mem had significantly fewer numbers of NFTs in hippocampal CA1 as compared to mice in the SH-sal control group. (SH-sal mice: 37.1±1.2; SH-mem mice: 24.7±1.77; EH-sal mice: 29.9±1.37; EH-mem mice: 13.2±1.23). The number of NFTs in EH-mem mice was significantly lower than that of SH-mem (*P* < 0.05) and EH-sal mice (*P* < 0.05); however, no significant difference was observed between SH-mem and EH-sal mice (*P* > 0.05) (***Fig. 2B***). The above results suggested that both memantine and EE dramatically reduced NFTs in SAMP-8 mice and combination of memantine with EE caused greater reduction in NFTs than either drug used alone.

### Western blotting of APP

Aβ levels can be affected by a variety of proteins involved in Aβ production. APP is the Aβ precursor whose level directly determines Aβ level in AD. Therefore, in this study, we attempted to examine the effect of memantine combined with EE on the level of APP in SAMP8 mice. Our measurements indicated that both memantine and EE significantly decreased APP levels in the hippocampus as compared to the standard housing control mice. Two-way ANOVA analysis indicated that the APP level in the hippocampus had a significant effect of memantine treatment (F_1,27_ = 7.37, *P* < 0.001) as well as EE (F_1,27_ = 4.66, *P* < 0.001) and a significant interaction between memantine treatment and EE (F_3,9_ = 2.99, *P* < 0.001). Multiple Bonferroni test showed that memantine combined with EE (0.141±0.022) significantly decreased APP levels compared to memantine (0.337±0.02) or EE alone (0.351±0.018) (*P* < 0.05). However, there was no significant difference in APP level between SH-mem and EH-sal mice (*P* > 0.05) (***Fig. 3A*** **and** ***3B***). The above APP Western blot data suggested that memantine combined with EE dramatically reduced AD-like pathology in SAMP-8 mice, which is consistent with the above immunohistochemistry results.

## DISCUSSION

The aim of this study was to determine whether combining two distinct therapies, memantine and EE, which have been shown to promote cognitive and pathological improvement when provided separately, would yield greater benefits. The effect of memantine and EE were evaluated alone and in combinaiton in a widely used AD model, SAMP8 mice. The data revealed that chronic administration of memantine or exposure to EE facilitated spatial learning and memory relative to the saline-treated controls, which is consistent with data from a very recent study[25]. Our data also showed that memantine treatment or EE exposure lessened hippocampal CA1 NFT accumulation and decreased the whole hippocampal APP levels, which are in agreement with previous reports[25]-[27].The combination paradigm of memantine and EE also demonstrated improved spatial learning and memory and decreased CA1 NFT accumulation and the whole hippocampal APP levels versus saline-treated standard housing controls. Moreover, our data showed that memantine and EE, when combined, demonstrated more learning and memory improvement and less AD-like pathology, including CA1 NFT accumulation and the whole hippocampal APP levels than either alone.

Glutamate is the most important excitatory neurotransmitter in the central nervous system. Physiological glutamate receptor activation is important for normal brain function whereas excessive glutamate receptor activation is thought to contribute to many neurological disorders ranging from acute hypoxic ischemic brain injury to chronic neurodegenerative diseases such as AD, Parkinson's and Huntington's disease[28]. Overactivation of NMDA subtype of glutamate receptor (NMDAR) and subsequent influx of excessive Ca^2+^ is the most common neurotoxicity in these diseases. Therefore, inhibition of excessive glutamate receptor activation may be a potential therapeutic strategy for neuroprotective actions. Memantine, an uncompetitive antagonist of NMDAR, has been approved by FDA to improve cognitive function in patients with moderate to severe AD[8]. The pharmacological action of memantine in AD is thought to be based on its ability to reduce overactivation of NMDA receptors, while allowing normal activity to occur. In this study, we found that memantine either alone or combined with EE improved spatial learning and memory and reduced AD-like pathology, including decreased NFT accumulation and lowered APP levels in the hippocampus.

The importance of hippocampal formation for memory function has been demonstrated in selective lesion experiments in animal studies[29],[30]. The hippocampus is central to the formation of new memories and memory consolidation[31]. The entorhinal cortex relays information from the sensory cortical regions to the hippocampus. This information are processed by the hippocampus and transferred to the permanent storage sites in the neocortex[32]. Dysfunction of these regions leads to impairment in several types of memory, including spatial and recognition memory and operant learning[29],[30]. NFTs, which are formed by hyperphosphorylation of the microtubule associated protein tau and Aβ, which is produced by APP, are the key neuropathological features in the brain of AD patients. All these features occur in an increasing number during the progression of AD, with prominence in the temporal neocortex, entorhinal cortex and hippocampus in the early phases[33]. Degenerative changes in these structures play a major role in memory dysfunction observed at early stages of AD[34],[35]. A very recent study implicated both amyloid deposition and tau pathology in the hippocampus as an early and late cause of decline in memory function over time in AD and memory performance appeared to be specifically related to the amount of amyloid plaques and NFTs in the entorhinal cortex and hippocampus[36]. The senescence-accelerated mouse (SAM) is a model of accelerated senescence that was established through phenotypic selection from a common genetic pool of AKR/J strain of mice[17]. Recently, SAMP8 has drawn considerable attention in AD research[37],[38]. SAMP8 mice, as a model of aging, display many additional features that are known to occur early in the pathogenesis of AD such as increased Aβ alterations, tau hyperphosphorylation, and a decrease in brain levels of acetylcholine[39]. Intracerebroventricular injection of Aβ antibody in 12 month old SAMP8 mice with a heavy load of Aβ and significant learning and memory impairment increased acetylcholine levels in the hippocampus[40]. SAMP8 mice are an excellent model for studying neurodegenerative changes associated with AD. Consistent with the above studies, we found that in saline-treated SH SAMP8 mice, there were higher levels of NFT accumulation and APP expression in the hippocampus, which is highly related to spatial learning and memory deficit. Many studies demonstrated that both memantine and EE have shown therapeutic effect on AD. Memantine treatment has been shown to restore cognition and significantly reduce the levels of insoluble Aβ and memantine treatment was also associated with a decline in the levels of total tau and hyperphosphorylated tau[25].

EE prevents cognitive decline and anxiety-related behavior in transgenic mice with AD-like pathology[41]–[44]. Additionally, it was reported that EE is able to reduce Aβ plaque burden and the extent of cerebral amyloid angiopathy[41],[44]–[46], probably mediated by induction of Aβ clearance and disrupted amyloid aggregation, utilizing increased activation of the Aβ degrading endopeptidase neprilysin[46], reduced inflammation and oxidative stress[45],[47], enhanced microglial phagocytosis[45] as well as boosted angiogenesis and modulated expression of Aβ receptor/transporter systems facilitating Aβ efflux across the blood brain barrier[48].

However, so far, there is no study on the therapeutic effect of combination of memantine and EE in AD. For the first time, we evaluated the potential additive benefit of memantine and EE and our results showed additive effect on learning and memory as demonstrated by significant shorter escape latencies and higher frequency of target entrance than either drugs used alone. Consistent with the behavior results, pathological studies showed that combining these two therapies synergistically lessened NFTs and APP expression as compared to either drugs used alone in SAMP8 mice, indicating that the combination of memantine with EE might be a novel and efficient therapeutic strategy for the treatment of AD.
